# Supplementary material for: Complex‐centric proteome profiling by SEC‐SWATH‐MS
Source: Mol Syst Biol. 2019 Jan 14;15(1):e8438. doi: 10.15252/msb.20188438 (PMC6346213; doi:10.15252/msb.20188438)
Supplement: Supplementary file 7 — Dataset EV6 [file MSB-15-e8438-s007.zip › feature_plots_bioplex/Q9Y5M8.pdf]

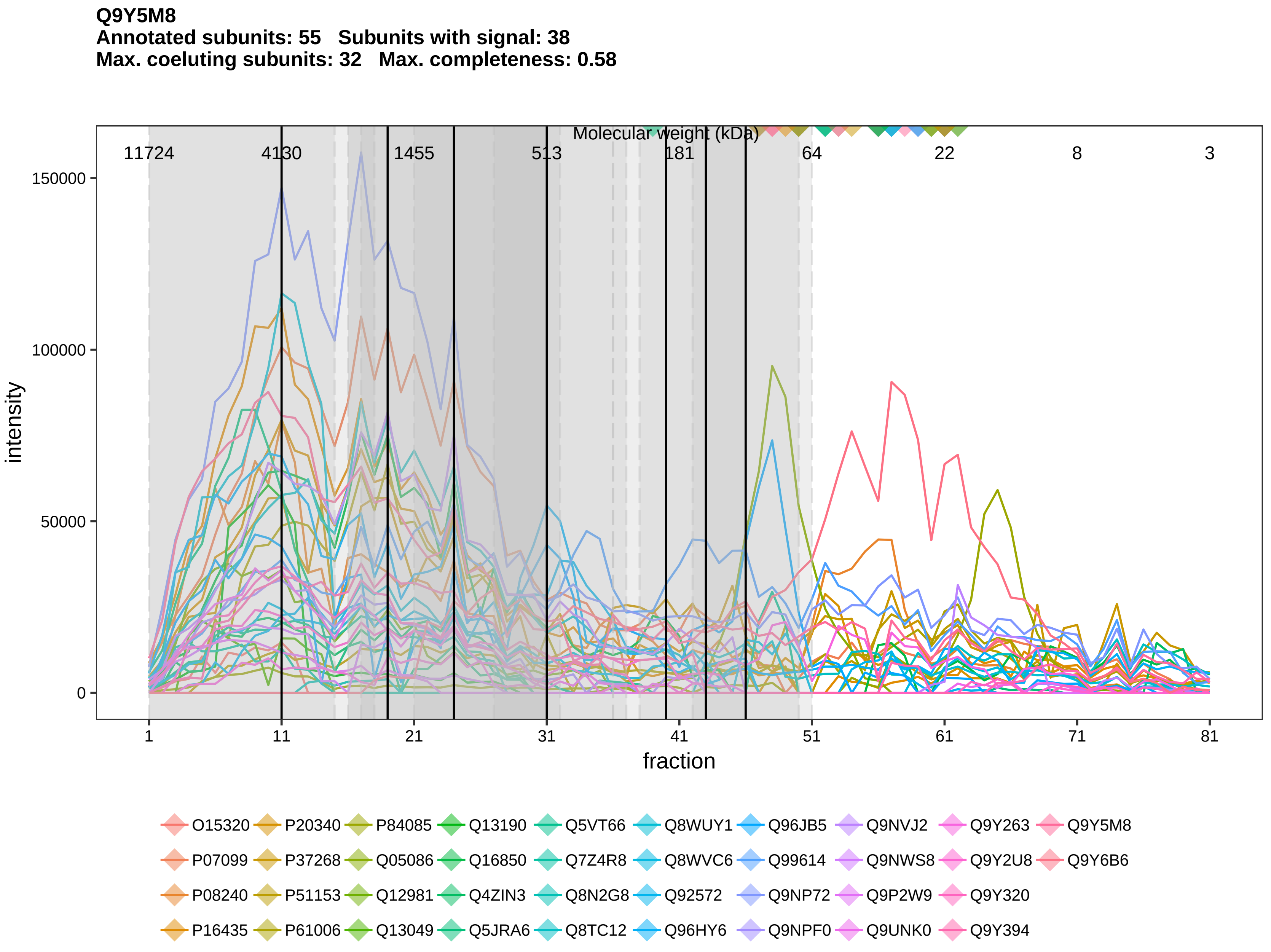

Q9Y5M8
Annotated subunits: 55 Subunits with signal: 38
Max. coeluting subunits: 32 Max. completeness: 0.58
Molecular weight (kDa)
11724
4130
1455
513
181
64
22
8
3
intensity
150000
100000
50000
0
fraction
1
11
21
31
41
51
61
71
81
O15320
P20340
P84085
Q13190
Q5VT66
Q8WUY1
Q96JB5
Q9NVJ2
Q9Y263
Q9Y5M8
P07099
P37268
Q05086
Q16850
Q7Z4R8
Q8WVC6
Q99614
Q9NWS8
Q9Y2U8
Q9Y6B6
P08240
P51153
Q12981
Q4ZIN3
Q8N2G8
Q92572
Q9NP72
Q9P2W9
Q9Y320
P16435
P61006
Q13049
Q5JRA6
Q8TC12
Q96HY6
Q9NPF0
Q9UNK0
Q9Y394
